# Morpho-Physiological, Yield, and Transgenerational Seed Germination Responses of Soybean to Temperature

**DOI:** 10.3389/fpls.2022.839270

**Published:** 2022-03-22

**Authors:** Firas Ahmed Alsajri, Chathurika Wijewardana, Raju Bheemanahalli, J. Trenton Irby, Jason Krutz, Bobby Golden, Vangimalla R. Reddy, K. Raja Reddy

**Affiliations:** ^1^Department of Plant and Soil Sciences, Mississippi State University, Mississippi, MS, United States; ^2^Field Crops Department, Tikrit University, Tikrit, Iraq; ^3^Mississippi Water Resources Research Institute, Mississippi State University, Mississippi, MS, United States; ^4^Delta Research and Extension Center, Stoneville, MS, United States; ^5^Adaptive Cropping Systems Laboratory, USDA-ARS, BARC-W, Beltsville, MD, United States

**Keywords:** gas exchange, reproductive growth, seed germination, soybean yield, temperature, transgenerational effects

## Abstract

Temperature is the primary factor affecting the morpho-physiological, developmental, and yield attributes of soybean. Despite several temperature and soybean studies, functional relationships between temperature and soybean physiology and yield components are limited. An experiment was conducted to determine the optimum temperature for soybean gas exchange and yield components using indeterminate (Asgrow AG5332, AG) and determinate (Progeny P5333 RY, PR) growth habit cultivars. Plants grown outdoors were exposed to 5 day/night temperature treatments, 21/13, 25/17, 29/21, 33/25, and 37°C/29°C, from flowering to maturity using the sunlit plant growth chambers. Significant temperature and cultivar differences were recorded among all measured parameters. Gas exchange parameters declined with increasing temperature treatments during the mid-pod filling stage, and quadratic functions best described the response. The optimum temperature for soybean pod weight, number, and seed number was higher for AG than PR, indicating greater high-temperature tolerance. Soybean exposed to warmer parental temperature (37°C/29°C) during pod filling decreased significantly the transgenerational seed germination when incubated at 18, 28, and 38°C. Our findings suggest that the impact of temperature during soybean development is transferable. The warmer temperature has adverse transgenerational effects on seed germination ability. Thus, developing soybean genotypes tolerant to high temperatures will help growers to produce high-yielding and quality beans. The quantified temperature, soybean physiology, and yield components-dependent functional algorithms would be helpful to develop adaptation strategies to offset the impacts of extreme temperature events associated with future climate change.

## Introduction

Soybean [*Glycine max* (L.) Merr.] is one of the most dominant oilseed crops in the world (124 M ha and 348.7 M tons),^1^ providing more than 29% of the oil and 71% of protein meal for human and livestock consumption (Assefa et al., 2019). The United States accounts for 32% of the global soybean production with an average yield of about 1,418 kg acre^–1^ (USDA-ERS, 2019). With the rapid increase in the global population, there is a need to increase soybean yield to keep pace with the growing demand under a rapidly changing climate. Among the changes projected in the environment, current and projected changes in temperatures are reported to have negative impacts on major crops, such as soybeans (Porter and Gawith, 1999; Wheeler and von Braun, 2013; Zandalinas et al., 2021).

The mean annual temperatures of soybean, corn, rice, and wheat growing areas have increased by 1°C during the past century (Zhao et al., 2017). Global climate models predict with high certainty that temperatures will increase by 1–4°C, depending on regions, by the end of the twenty-first century (Intergovernmental Panel on Climate Change [IPCC], 2018). A slight increase in seasonal and diurnal changes in temperature during the whole season or around flowering and post-flowering stages negatively impacts the yield and quality of many crops (Reddy et al., 1992, 1997; Bheemanahalli et al., 2016, 2019; Prasad et al., 2017). If high temperatures coincide with crops flowering and grain-filling stages, reduction in yield will be severe due to the impaired morpho-physiological and pollen vitality (Djanaguiraman et al., 2013, 2019; Prasad et al., 2017; Cohen et al., 2021). The increasing high-temperature episodes threaten the soybean production (Cohen et al., 2021; Zandalinas et al., 2021). For every 0.8°C increase above 26.7°C, the current mean temperature of the southern United States, soybean yields are expected to decline by 2.4% (Hatfield et al., 2011). Therefore, understanding globally significant oil and protein crop, such as soybean responses to temperature is essential to provide the functional database needed to improve the crop models and develop mitigation strategies to alleviate such changes.

High temperature negatively impacts the soybean reproductive processes and seed set (Koti et al., 2007; Salem et al., 2007; Djanaguiraman et al., 2013, 2019). However, the impact of high temperature varies with duration, the intensity of stress, and cultivars (Sionit et al., 1987; Schlenker and Roberts, 2009). Soybean yield declined by 17% with a 1°C increase in temperature from its optimum across soybean growing areas (Lobell and Asner, 2003; Lobell et al., 2011). Similarly, 1–3°C increase in temperatures from its optimum, reduced the number of pods per plant (10–30%), number of seed (11–35%), seed size (5–14%), and seed yield (16–40%) (Tacarindua et al., 2013). Along with reproductive and grain filling events, the soybeans’ physiological and biochemical processes are also sensitive to high-temperature stress. With a 4°C increase in temperature from 22/16°C, net photosynthesis was increased by 59% (Sionit et al., 1987). Soybean plants grown at 38/28°C showed considerable reduction (19.7%) in photosynthesis rate compared with the optimum temperature (28/18°C) after 14 days of treatment during the R2 stage (Djanaguiraman et al., 2011a). An increase in temperature during the reproductive phase decreased photosynthesis by 22%, associated with an 11% reduction in pod-set (Djanaguiraman et al., 2019). To our knowledge, there is limited evidence on the functional relationship between temperature and gas exchange parameters during post-flowering in soybean.

Previous reports have shown that stress adaptive responses are inherited through plant transgenerational memory. Substantial progress in understanding transgenerational response to abiotic stresses is mainly reported in model species or few crops. It was reported that maternal growing conditions (water-deficit treatments) negatively influenced the seedling growth and development in soybean (Wijewardana et al., 2019). To date, little or no information is available on the effects of gradient temperatures on transgenerational seed germination on soybean. Here, we hypothesized that two soybean cultivars respond to five different temperatures differentially during the post-flowering stage, and temperature effects are transferable to the next generations. The objectives of this study were to (i) quantify the effect of gradient temperatures on gas exchange parameters, (ii) quantify the temperature effects on yield components of two soybean cultivars, (iii) determine the effect of temperatures on the transgenerational seed germination, and (iv) to provide quantitative information that could be useful to improve the functionality of soybean crop models for field applications.

## Materials and Methods

### Experiment 1

#### Crop Husbandry and Temperature Treatments

Two soybean cultivars, Asgrow AG5332 (AG) and Progeny P5333RY (PR) from maturity group V (MG-V) were used in the study. These cultivars are recommended ideal for the US Midsouth environments. Four treated seeds were sown in polyvinyl chloride (PVC) plastic pots (15.2 cm diameter and 30.5 cm height). The pots were filled with the soil medium of 3:1 sand: topsoil classified as sandy loam, 2% clay, 11% silt, and 87% sand, with 0.5 kg of pea gravel at the bottom of each pot. Each pot had a hole at the bottom to drain the extra water. Plants were thinned to one, 7 days after emergence. Pots were arranged outdoors on two benches, one bench for each cultivar until initial flowering.

**FIGURE 1:**
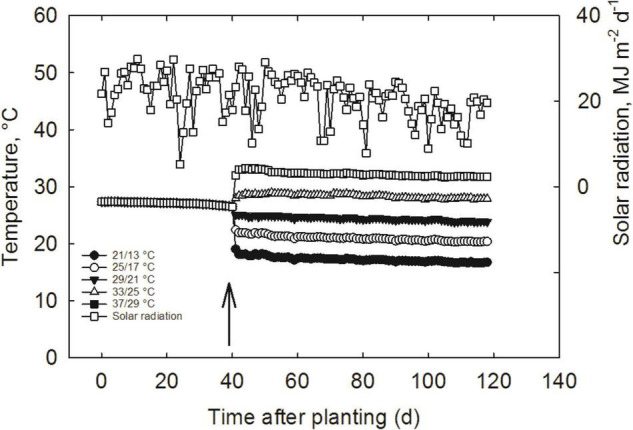
The schematic presentation of observed mean daily air temperature and solar radiation levels during the experiment. The upward arrow on the panel indicates the date on which temperature treatments were imposed.

The experiment was conducted in the Soil-Plant-Atmosphere-Research (SPAR) facility, the sunlight environmental growth chambers at the Environmental Plant Physiology Laboratory, Mississippi State University, United States. The specifications and operation of SPAR units have been detailed in our previous report (Reddy et al., 2001). Plants were moved to SPAR chambers just before the initial flowering stage. In each SPAR, 12 plants from the same cultivars were arranged randomized, five SPARs were used for each cultivar, and overall, ten SPARs were used for the experiment. Five day/night temperature treatments, 21/13 (actual: 21.2/13.4), 25/17 (actual: 24.8/17.2), 29/21 (actual: 28.3/20.6), 33/25 (actual: 32.1/24.4), and 37°C/29°C (actual: 35.7°C/28.4°C, day/night), were imposed at flowering and continued until maturity (Figure 1 and Table 1). Air temperature in each chamber was monitored and adjusted every 10 s throughout the day and night and maintained within ± 0.5°C of the treatment set points measured with aspirated thermocouples. Plants were irrigated three times a day through an automated and computer-controlled drip system with full-strength Hoagland’s nutrient solution (Hoagland and Arnon, 1950), delivered at 07:00, 12:00, and 17:00 h, to ensure balanced nutrient and moisture conditions for plant growth. All SPARs were maintained at 400 ppm (CO_2_) until harvest.

**TABLE 1:** The set treatments and observed mean of the daily measured chamber (CO_2_), temperature, vapor pressure deficit (VPD), and evapotranspiration (ET) during the experimental period.

Set treatments	Measured parameters			
Day/night temperature	Mean temperature	Chamber CO_2_ concentration	Mean daily VPD	Mean daily ET
**°C**		**ppm**	**kPa**	**L** **m^–2^** **d^–1^**

21/13	17.3e*	418a	0.75e	9.82c
25/17	21.0d	417a	1.14d	8.95c
29/21	24.4c	421a	1.59c	11.95b
33/25	28.2b	419a	2.35b	11.52b
37/29	32.1a	421a	3.00a	14.61a

*Values in each column followed by the same letter are not significantly different (p < 0.05) according to Fisher’s least significant difference (LSD).

Throughout this experiment, the received daily solar radiation was recorded with a pyranometer (Model 4-8; The Eppley Laboratory Inc., Newport, RI). The relative humidity of each SPAR chamber was monitored with a temperature and humidity sensor (HMV 70Y, Vaisala Inc., San Jose, CA, United States) placed in the path of airline ducts. The CO_2_ concentration in each SPAR chamber was adjusted and monitored every 10 s during the day and maintained at 400 ppm throughout daylight hours using a dedicated LI-6250 CO_2_ analyzer (Li-COR, Inc., Lincoln, NE, United States). Variable density shade cloths were designed and placed around the edges of the plant canopy and adjusted regularly to match the plant’s canopy height and exclude the need for border plants. The vapor pressure deficits (VPD) in the treatment were estimated from these measurements as per Murray (1967; Table 1). Evapotranspiration (ET) rates were measured on a ground area basis (L m^–2^ d^–1^) throughout the treatment period and calculated as the rate at which condensate was removed by the cooling coils at 900 s intervals (McKinion and Hodges, 1985; Reddy et al., 2001) by measuring the mass of water in collecting devices connected to a calibrated pressure transducer (Table 1). Except for temperature treatments, all SPARs have the same growth conditions, and cultivars were completely randomized within each SPAR.

#### Gas Exchange Measurements

Gas exchange parameters were measured using the LI-6400 photosynthesis system (LiCOR Inc., Lincoln, NE, United States). The instrument was set to 400 ppm CO_2_ concentration, 1,500 μmol m^–2^ s^–1^ light intensity (PAR), and the temperature was set to similar to the treatment daytime temperatures as, 21, 25, 29, 33, or 37°C. Relative humidity was set at a near ambient level (50%). The measurements were made on each plant’s uppermost third fully expanded leaf at 27 days after treatment (DAT) between 10:00 and 12:00 h. The flow rate through the chamber was adjusted to 350-mol s^–1^. Photosynthesis was recorded as the total coefficient of variation (%CV) reached a value of less than 0.5. The instrument calculates stomatal conductance and transpiration by considering leaf area and incoming and outgoing flow rates. Intrinsic water use efficiency (WUE) was estimated as the ratio of photosynthesis to transpiration.

#### Yield and Yield Components

All plants were harvested 120 DAP (82 DAT). The number of pods plant^–1^ was recorded at the time of harvest. Roots were separated from the shoot, and those were placed on a sieve size 1 m × 1 m and 1.5-m high and washed gently using a gentle water stream. Stem, leaf, and root dry weights were recorded by placing those materials in a forced-air dryer set at 75°C for 72 h, and the pod dry weight was taken after drying at 25°C for 7 days. Seeds were separated from the pods manually, and dry weight was taken. Seed count was taken using Old Mill Seed Counter (NP5056-Model 850-2, LICOR Inc., Lincoln, NE, United States). Whole-plant dry weight was calculated by adding stem, leaf, and root dry weights after weighing each component. The harvest index was calculated by dividing the seed yield by the whole plant biomass.

### Experiment 2

#### Germination of Seeds Exposed to Parental Temperature

Harvested seeds from the Exp. 1 were then exposed to three different incubation temperatures (18, 28, and 38°C) in Exp. 2 to study the parental temperatures induced changes in the seed germination of offspring. Four replicates of 100 matured seeds from each paternal temperature were germinated using the incubator (Thermo Fisher Scientific, Marietta, OH, United States). The incubator was set at 18, 28, and 38°C, considered low, optimum, and high temperatures for seed germination, respectively. After 24 h of incubation, the number of seeds germinated was recorded at 2-h intervals until 3 days after incubation. As the germination rate slowed down, observation intervals were extended 4, 6, and 12 h. Once germination ceased for 3 consecutive days, the trial was terminated. The Watchdog Model 100 data loggers (Spectrum Technologies Inc., Aurora, IL, United States) were used to record the actual temperature during the treatment period. To evaluate the transgenerational effects of temperature stress on the progeny fitness, *in vitro* seed germination parameters, such as maximum seed germination, time to 50% seed germination, and seed germination rate were measured.

### Statistical Analysis

This study was treated as a completely randomized design for statistical analysis purposes. Proc ANOVA analysis procedure (ANOVA) was performed on the replicated values of the measured parameters using the PROC general linear model (GLM) procedure in SAS (SAS Institute Inc, 2011, Cary, NC, United States) to test the significance of temperature, cultivars, and their interactions on all measured and calculated traits. Fisher’s-protected least significant difference (LSD) tests at *p* ≤ 0.05 were used to determine the significance of temperature and cultivar’s effect. The germination time course was fitted to a three-parameter sigmoidal function similar to a procedure described in other studies (Wijewardana et al., 2019; Walne et al., 2020) using the Sigma Plot 14.5 (Systat Software Inc., San Jose, CA, United States). Maximum seed germination and time to 50% seed germination responses to the parental temperature were estimated for each *in vitro* germination temperature. The regression and graphical analysis were performed using Sigma Plot 14.5 (Systat Software Inc., San Jose, CA, United States) to determine the best-fit equations to describe the relationship between soybean cultivars, traits, and temperature.

## Results and Discussion

The experimental conditions indicated no significant differences between the two SPARs having the same temperature condition; however, there were substantial differences among the temperature treatments (Figure 1). Average day/night temperatures used in the study represent the variability we expect across the global and the United States soybean growing belts during the flowering and pod-filling stages. On average, all the temperature treatments were on par with the target temperatures (Table 1 and Figure 1). The daily solar radiation ranged from 5.27 to 29.83 MJ m^–2^ day^–1^ with an average of 20.06 MJ m^–2^ day^–1^ (Figure 1) that typically far exceeds the light levels conducted under indoor plant growth chambers (Djanaguiraman et al., 2013). At the same time, the VPD was 2.3 kPa lower at the low temperature (17.3°C) compared with a higher temperature (32.1°C). Observed linear increase VPD with air temperature indicated that other weather variables might play a significant role in plant performance (Kobayasi et al., 2010; Grossiord et al., 2020). The measured ET showed 9.82 and 14.61 L m^–2^ day^–1^ for the low and high temperatures, respectively (Table 1).

### Responses of Gas Exchange Parameters to Temperature

Temperature treatments during reproductive growth stages showed a substantial effect on all gas exchange traits (Table 2). Further, 27 days after treatments, most gas exchange traits significantly changed between the two cultivars (Table 2). The interaction between temperature and cultivar had no effect on photosynthesis and transpiration (Table 2). Extreme temperatures significantly affected gas exchange traits, with photosynthesis, stomatal conductance, and transpiration being significantly lower at 17.3°C than 28.2°C.

**TABLE 2:** Analysis of variation across the temperatures (T), cultivars (C), and their interaction (T × C) with morphological and physiological traits.

Parameters	T	C	T × C
Photosynthesis (μmol m^−2^ s^–1^)	***	***	ns
Stomata conductance (mol m^–2^ s^–1^)	***	***	**
Transpiration rate (mmol H_2_O m^–2^ s^–1^)	***	***	ns
Water-use efficiency (mmol CO_2_ mol^–1^ H_2_O)	***	ns	**
Flowering to maturity (R1 to R8) (days)	***	***	***
Total plant dry weight (g plant^–1^)	*	ns	***
Root dry weight (g plant^–1^)	ns	***	***
Stem dry weight (g plant^–1^)	***	***	***
Leaf dry weight (g plant^–1^)	***	***	**
Pod numbers (no. plant^–1^)	***	***	***
Seed numbers (no. plant^–1^)	***	***	**
Pod dry weight (g plant^–1^)	***	***	**
Seed dry weight (g plant^–1^)	***	***	**
Harvest index (plant basis)	***	***	**

*Gas exchange parameters were measured 65 days after planting and 27 days after treatment. Flowering to maturity is measured by observing events. The other traits were measured at the final harvest, 120 days after sowing, and 82 days after treatment.*

*, **, and *** indicates a significance at p = 0.05, 0.01, and 0.001 level, respectively, and ns indicated non-significance.

**FIGURE 2:**
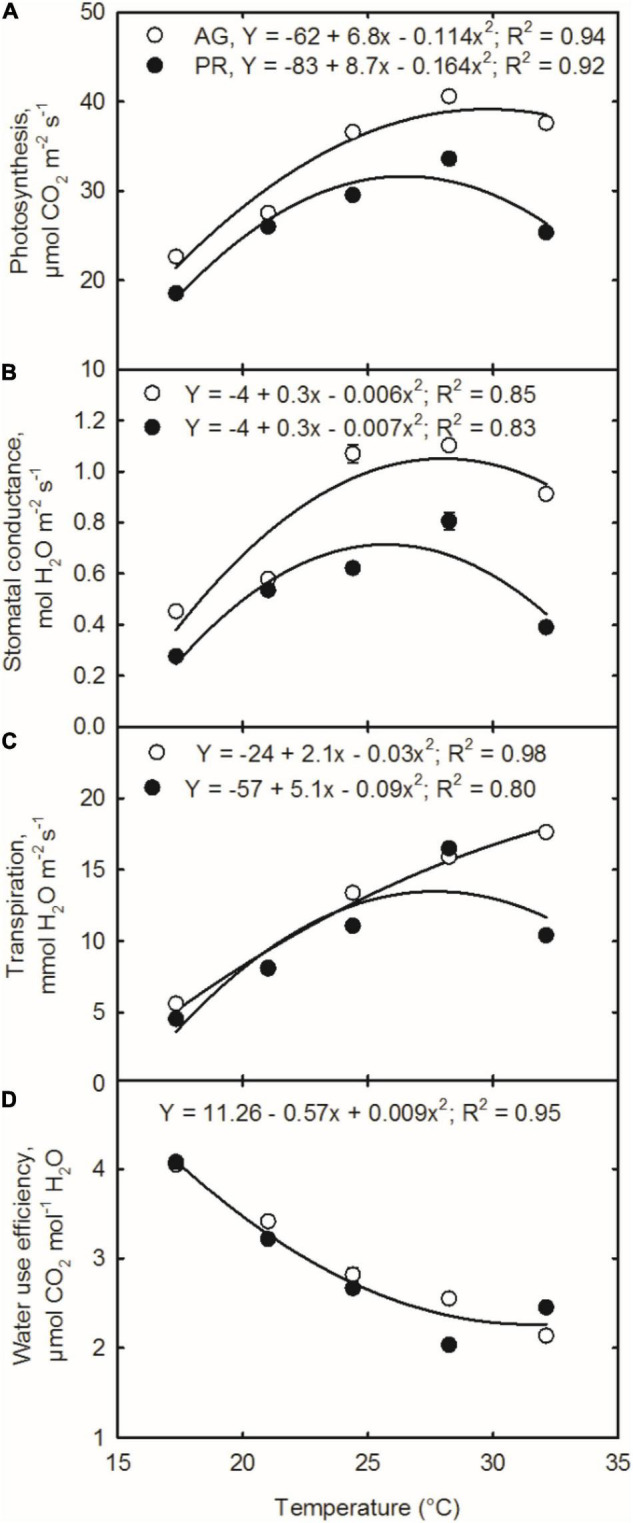
Temperature effects on **(A)** photosynthesis, **(B)** stomatal conductance, **(C)** transpiration rate, and **(D)** water using efficiency of soybean cultivars [Asgrow AG 5332 (AG) and Progeny P5333 RY (PR)]. Measurements were taken 65 days after sowing and 27 days after temperature treatments. SEM ± four observations are presented if the values are larger than the symbol size.

Cultivar AG exhibited higher photosynthesis potential than PR cultivar, and maximal photosynthesis was recorded at 28.2°C (Figure 2A). Photosynthesis was reduced by 44, 32, 10, and 7% for AG and 45, 23, 12, and 25% for PR at 17.3, 21, 24.4, and 32.1°C temperature treatments, respectively, compared with the plants grown at 28.2°C (Figure 2A). For both the cultivars, photosynthesis increased with increasing temperature until reaching thermal optimal (28.2°C) and then decreased with further increase in temperature (Figure 2A). Stomatal conductance has a varied response with temperature treatments and followed a quadratic response to temperature (Figure 2B, *R*^2^ = 0.85 and 0.83 for AG and PR, respectively). Across cultivars, the stomatal conductance was maximum in plants grown at 28.2°C (Figure 2B). Stomatal conductance decreased by 59 and 18% for AG and 65 and 52% for PR when soybean plants were grown under low (17.3°C) and high (32.1°C) temperatures, respectively, compared with the plants grown at 28.2°C (Figure 2B). Overall, AG cultivar exhibited increased stomatal conductance than PR across all temperature conditions. Since soybean is a warm-season crop, the plants grown in sub-optimum temperature had the highest reduction in photosynthesis compared with other temperature treatments. Our findings suggest that photosynthesis is hindered when plants grow under low temperatures with high solar radiation conditions. The low-temperature-induced photoinhibition and modifications to the membrane thylakoid might have caused the lower photosynthetic rate (Moon et al., 1995). Low temperature decreases Rubisco performance and cell activity and reduces the root water absorption (Zhu, 2001; Zhao et al., 2004) and limiting photosynthesis. Similar photosynthesis responses were noted in soybean under low temperatures (Taylor and Rowley, 1971; Caulfield and Bunce, 1988; Wang et al., 1997). On the other hand, at the high temperature, reduced chlorophyll content, impaired function of PSII, and decreased electron transport rate (Djanaguiraman et al., 2011a,^2013^) might have caused damage to the photosynthetic machinery (Gibson and Mullen, 1996; Zhang et al., 2016). However, an increase in stomatal conductance increased the photosynthetic rate (*R*^2^ = 0.94, *p* < 0.001), and thus, the relationship holds across a range of growing temperature conditions (Supplementary Figure 1).

Temperature and cultivar significantly affected the transpiration rate and WUE (Table 2 and Figures 2C,D). In cultivar AG, transpiration was highest in plants grown at the highest temperature, 32.1°C. Whereas, in PR, the transpiration was the greatest at 28.2°C (Figure 2C). On average, the AG cultivar had a 20% higher transpiration rate than the PR cultivar when averaged across temperature conditions. Surprisingly, irrespective of treatments, WUE remained almost constant between cultivars (Figure 2D). Increased temperature treatments reduced WUE by 19, 33, 44, and 44% for plants grown at 21, 24.4, 28.2, and 32.1°C treatments, respectively, compared with those at low temperatures, 17.3°C (Figure 2D). The highest WUE of soybean at 17.3°C treatment is associated with reduced stomatal conductance (Figure 2) and photosynthesis, probably through reduced Rubisco activity. Though there were no significant differences between the cultivars for internal CO_2_ concentration (∼306 ppm), the reduction in photosynthesis could be attributed to the stomatal mechanisms under extreme temperatures. At the highest temperature treatment, i.e., at 32.1°C, photosynthesis and transpiration are reduced because of the limiting stomatal conductance (Figure 2B). On the other hand, a decline in WUE at high temperatures might have caused by the combination of stomatal limitation and irreversible membrane degradations at the cell level (Moore et al., 2021). Higher temperature disrupts metabolic processes, such as the stomatal behavior and photosynthesis of plants (Mathur et al., 2014). A decline in photosynthesis found depends on the thermal optimal Rubisco enzyme and substrate regeneration, such as ribulose bisphosphate (RuBP) under high temperature (Feller et al., 1998; Sage et al., 2008). Differences in photosynthetic demand and parts can be explained by differences in leaf hydraulic and leaf respiration rate in responses to extreme temperatures (Cochard et al., 2000; Moore et al., 2021). It often complicates gas exchange processes because temperature change alters the VPD, which interferes with transpiration and WUE. However, at increased temperatures, principal factor controlling the photosynthesis is unclear.

### Responses of Soybean Yield Components to Temperature

**FIGURE 3:**
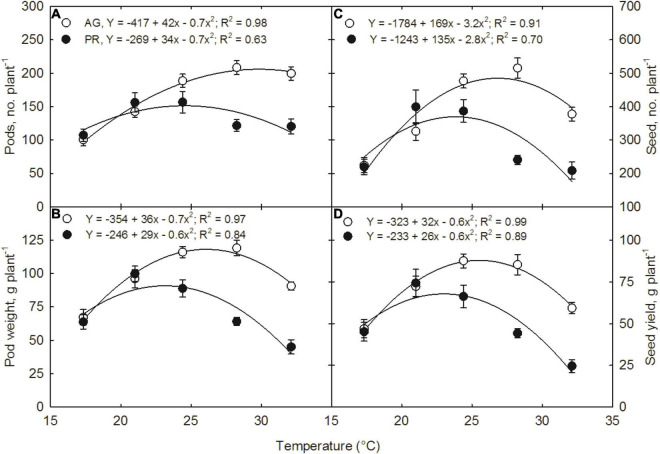
Temperature effects on **(A)** pods number, **(B)** pods dry weight, **(C)** seed number, and **(D)** seed yield of soybean cultivars, AG with indeterminate, and PR with determinate growth habits, respectively. Measurements were taken at 120 days after sowing and 82 days after temperature treatment. SEM ± 12 observations are presented if the values are larger than the symbol size.

**FIGURE 4:**
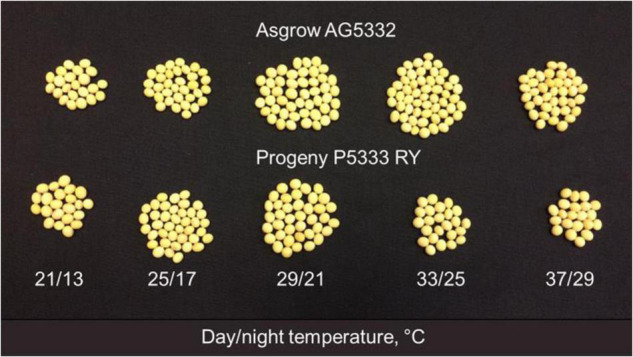
Temperature effects on a soybean seed number and size of AG and PR. The photograph represent 10% of the seed number per plant in each treatment. For total number of seed per plant, refer to Figure 3C.

**FIGURE 5:**
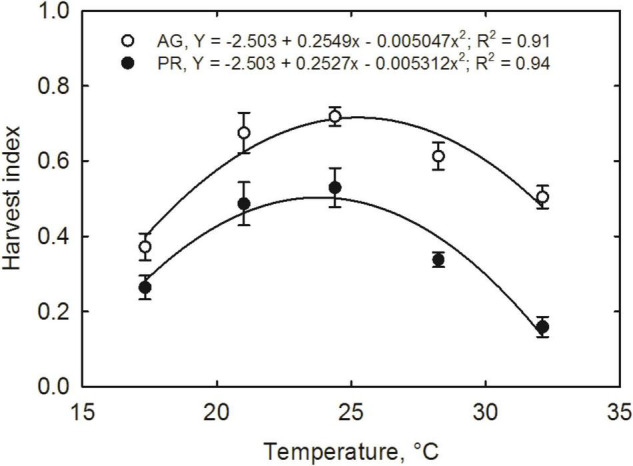
Temperature effects on the harvest index of soybean cultivars, AG with indeterminate, and PR with determinate growth habits, respectively. Measurements were taken at 120 days after sowing and 82 days after temperature treatment. SEM ± 12 observations are presented if the values are larger than the symbol size.

Increasing temperatures induced a substantial reduction in yield and yield components (Table 2). Pods number per plant increased quadratically with increasing temperature in both the cultivars (Figure 3A). The maximum pods per plant were observed at 28.2°C in AG and 24.2°C in PG. The decline in pod numbers at high temperatures was highest in PR (36.5 pods per plant) than the decline in AG (8.8 pods per plant) from the respective optimum temperature values (Figure 3A). Pod weight (Figure 3B), seed number (Figure 3C), and seed yield (Figure 3D) showed similar quadratic responses in both the cultivars in response to temperature treatments. The optimum temperatures for each yield-related trait varied slightly among the cultivars, 26 and 23°C for AG and PR, respectively, for pod dry weight, 27 and 24°C for AG and PR for seed number, respectively. At 24.2°C, the AG cultivar produced the maximum seed yield (88 g plant^–1^), whereas the PR cultivar recorded the maximum yield (44 g plant^–1^). On average, AG cultivar produced a greater yield across temperatures than PR cultivar (Figures 3, 4). Temperature treatments induced a significant reduction in 100-seed weight in both cultivars, with the lowest at 17.3^°^C (6%) and highest at 32.1°C (63%) in PR compared with 21°C. Previous studies have reported that warmer temperature reduced the pod set, seed number, and seed weight (Sionit et al., 1987; Salem et al., 2007; Djanaguiraman et al., 2013) in soybean. However, our results showed a more significant effect of higher temperatures on seed size or seed weight than seed number. The quadratic response functions best described the relationship between harvest index and temperature in both the cultivars (*R*^2^ = 0.91 for AG and *R*^2^ = 0.94 for PR). The harvest index increased with the rise in growing temperature up to 25°C in AG and 24°C in PR cultivars and then declined with a further increase in temperature (Figure 5). On average, cultivar AG exhibited a higher harvest index across all temperature treatments compared with PR. The decline at higher temperatures was steeper in PR (0.48 for 1°C^–1^) than in AG (0.28 for 1°C^–1^) cultivar showing the temperature sensitivity between the cultivars.

Most previous studies related to temperature effects at reproductive growth stages were conducted in the growth chambers with the unrealistic light source (Djanaguiraman et al., 2011b,^2013^). Unlike other controlled-environment studies, the SPAR systems have the advantage of precisely controlling air temperature, CO_2_ concentration, and air humidity under natural solar radiation compared with other controlled-environment facilities (Allen et al., 2003). Therefore, the growth and yields reported in this study are three times more than those reported under light-limited conditions (Djanaguiraman et al., 2011b,^2013^). The functional relationships developed between temperature and various growth and developmental processes, such as seed yield, could improve the existing soybean models (Boote et al., 2003; Nendel et al., 2011; Battisti et al., 2017). The study’s relationships and responses can be used for field applications and the policy arena (Liang et al., 2012). Therefore, the functional relationships between soybean growth and yield-related parameters data are more portable to field conditions and would be valuable to improve crop models for management and policy decisions.

### Warmer Temperature-Induced Transgenerational Changes in Seed Germination

To ascertain the impact of growth temperatures during grain filling, we quantified the *in vitro* germination of seeds collected from parental growth temperatures by germinating them under different incubation temperatures (18, 28, and 38°C). *In vitro*, seed germination parameters, such as cumulative seed germination, maximum seed germination, and time to 50% germination were significantly affected by parental temperature (*p* < 0.001) and incubation temperature (*p* < 0.001) (Table 3). Similarly, the interaction between cultivar and incubation treatment, growth temperature, and incubation treatment, cultivar × growth temperature differed significantly for germination traits, except cultivar × growth temperature for germination rate (Table 3). The significant three-way interaction of temperature × cultivars × growth temperature indicated that the parental temperature had a significant (*p* < 0.001) impact on maximum seed germination, but the magnitude of effects varied with the genotype.

**TABLE 3:** Analysis of variation across the temperatures, parental growth temperature, cultivars, and their interaction with maximum seed germination, time to 50% seed germination, and seed germination rate.

Sources of variation	Maximum seed germination	Time of%50 germination	Germination rate
Cultivar	ns	*	*
Growth temperature	***	***	***
Incubation temperature	***	***	***
Temperature × cultivars	**	***	***
Temperature × growth temperature	***	***	***
Cultivar × growth temperature	**	**	Ns
Temperature × cultivar × growth temperature	***	ns	Ns

*, **, and *** indicate significance at 0.05, 0.01, and 0.001 probability levels, respectively, and ns indicate non-significance.

**FIGURE 6:**
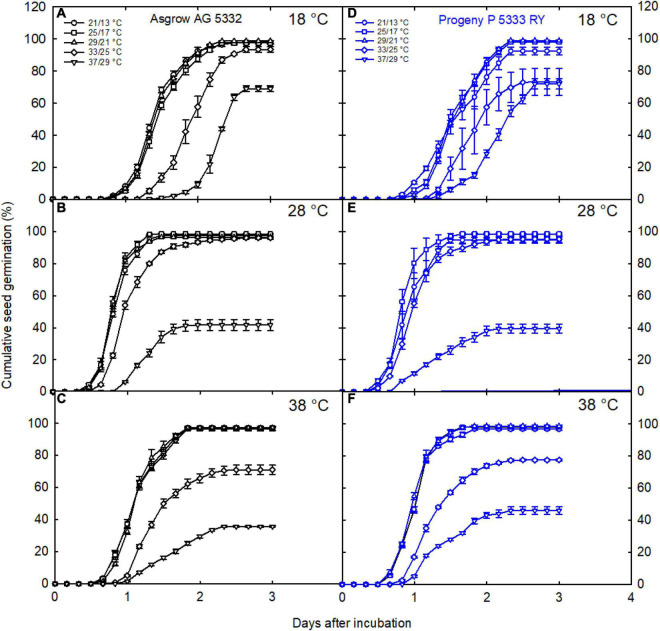
Variation in germination dynamics of seeds exposed to a range growing temperatures during grain filling. Seeds harvested from plants grown under five growing temperatures (21/13, 25/17, 29/21, 33/25, and 37°C/29°C) were *in vitro* germinated at incubation temperatures of 18, 28, and 38°C for 3 days to measure germination potential in AG **(A–C)** and PR **(D–F)** cultivars. The cumulative germination data of each genotype is presented using symbols in response to three incubation temperatures. The germination time course is represented by fitting a three-parameter sigmoidal function to each dataset.

**FIGURE 7:**
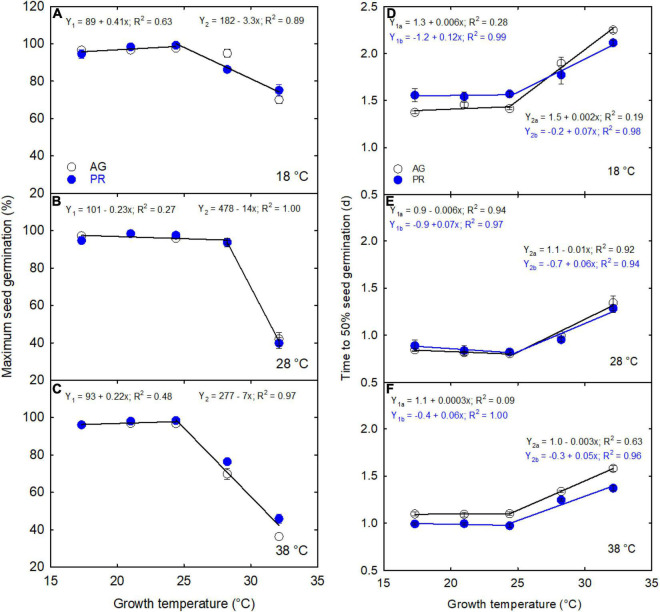
The effects of parental growing temperatures on next-generation maximum seed germination **(A–C)** and time to reach 50% germination **(E–F)** of AG and PR cultivars.

Soybean seeds produced under extreme growth temperatures (17.3 and 32.1°C) significantly decreased seed germination in both the genotypes and took more time to germinate in three incubation temperatures than 24.4°C (Figure 6). For instance, seeds collected from plants grown at 37/29°C were exposed to different incubation temperatures, such as 18, 28, and 38°C. At 48 h after incubation, about 18.8% (9.2% in AG and 29% in PR), 39.8% (41.7% in AG and 38% in PR), and 36.5% (29.5% in AG and 43% in PR) of cumulative seeds germinated under 18, 28, and 38^°^C, respectively (Figure 6). On the other hand, high temperature during pod filling affected the seed germination rates of both genotypes across three incubation treatments, as cumulative germination percentage was not 100% after 3 days of treatment (Figures 7A–F). Irrespective of cultivars, seeds harvested from the plants exposed to low and high temperatures, germinated (time to 50% germination) significantly later than the non-stressed seeds under low- and high-incubations temperatures (Figures 7D–F). Seeds with reduced weight showed poor seed germination under extreme incubation temperatures. For example, the seeds produced under high temperature (37/29°C) showed lower cumulative germination than 29/21°C across three incubation treatments (Figure 6). The percentage germination of seeds exposed to different growing temperatures varied 24 and 42 h after incubation (Figure 7). Seeds produced under hotter climates negatively affected the germination performance of the next generation when germinated under low and high temperatures (Figures 7A,C).

Accelerated seed development events, reduced size, and quality compositions were the most significant concern of heat stress during soybean pod development (Alsajri et al., 2020). Most studies reported the heat stress-induced changes in seed development and yield decrease in the legumes (Prasad et al., 2002; Sehgal et al., 2018). For example, the starch content of developing soybean seeds decreased when exposed to higher temperatures, suggesting the insufficient supply of resources to the sink or starch synthesis in seeds. However, biochemical and molecular pathways controlling these processes need to be investigated. Here, we hypothesize that extreme temperatures during pod filling reduce the assimilate production (photosynthesis) and supply to sink due to accelerated senescence, which negatively influences the seed size and quality compositions. Here, our study revealed the influence of parental temperature conditions on next-generation seed germination. These findings suggest that extreme temperatures alter the level and sensitivity of seeds phytohormone and enzymes, which regulate the subsequent generation of seed germination (Shi et al., 2016; Begcy et al., 2018). Furthermore, under high temperatures, the process of sink developmental and maturity events strongly influences the next-generation fitness in soybean, as shown under drought stress (Wijewardana et al., 2019). Our study shows that extreme growing temperatures during the pod development have a notable transgenerational effect on the seed germination of the soybean progeny.

## Conclusion

In this study, temperature effects on soybean physiology, growth, and yield parameters were quantified under optimum water, radiation, and nutrient conditions using the sunlit plant growth chambers. Temperature affected the physiological and developmental responses of soybean cultivars. The temperature optima varied among the cultivars for many parameters. In general, the indeterminate cultivar, AG, showed greater tolerance and high-temperature optimums for many traits suggesting that cultivars with indeterminate growth habits would be helpful under early planting production system in the Mid-South United States. In addition, temperature effects during the growth period will be transferable to the next generation affecting seed germination vitality traits. The transgenerational studies indicate that soybean seed produced in areas where growing temperatures are between 21 and 28°C would perform better across a wide range of temperature conditions in the next season. Improved models with appropriate process-level functional algorithms would be helpful in the management of natural resources and policy decisions both in the present and future warmer climate.

## Data Availability Statement

The original contributions presented in the study are included in the article/Supplementary Material, further inquiries can be directed to the corresponding author.

## Author Contributions

FA and KR contributed to the conception and design of the work. FA, CW, and KR collected the data. FA, RB, and KR contributed to the data analysis and interpretation. FA, CW, RB, JI, JK, BG, VR, and KR contributed to the article’s critical revision. KR gave final approval of the version to be published. All authors contributed to the article and approved the submitted version.

## Conflict of Interest

The authors declare that the research was conducted in the absence of any commercial or financial relationships that could be construed as a potential conflict of interest.

## Publisher’s Note

All claims expressed in this article are solely those of the authors and do not necessarily represent those of their affiliated organizations, or those of the publisher, the editors and the reviewers. Any product that may be evaluated in this article, or claim that may be made by its manufacturer, is not guaranteed or endorsed by the publisher.

## Footnotes

1
http://faostat.fao.org/
