# Supplementary material for: Morpho-Physiological, Yield, and Transgenerational Seed Germination Responses of Soybean to Temperature
Source: Front Plant Sci. 2022 Mar 22;13:839270. doi: 10.3389/fpls.2022.839270 (PMC8981302; doi:10.3389/fpls.2022.839270)
Supplement: Supplementary file 1 [file Data_Sheet_1.docx]

Supplementary Material


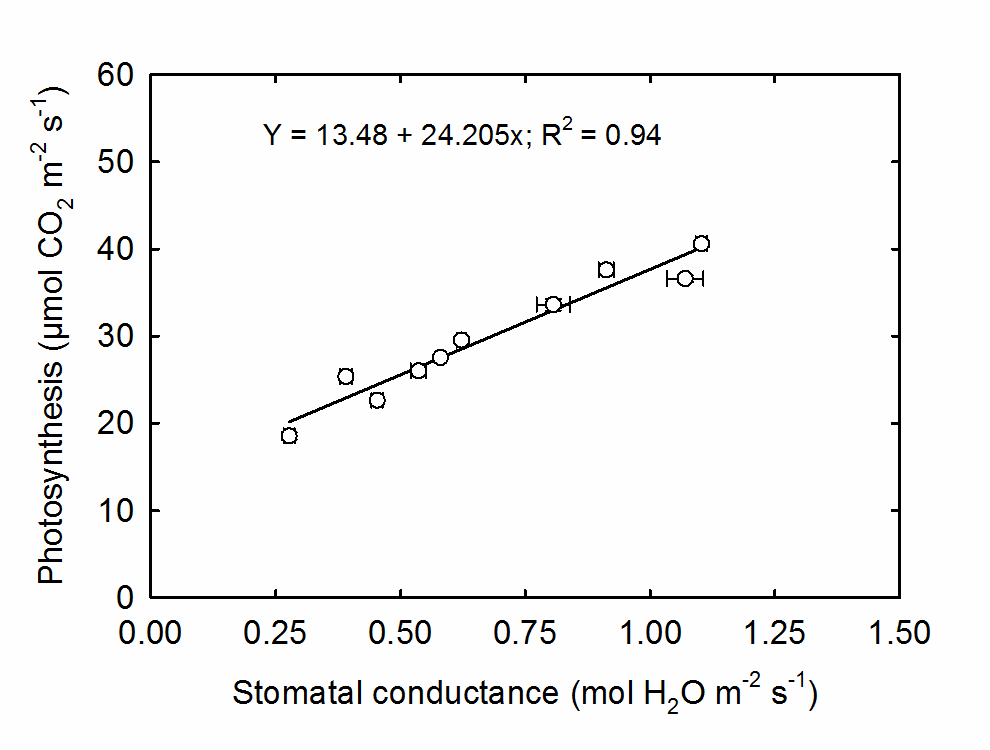


**SUPPLEMENTARY FIGURE 1** **|** The relationship between stomatal conductance and photosynthesis from the pooled data from cultivars and temperatures.


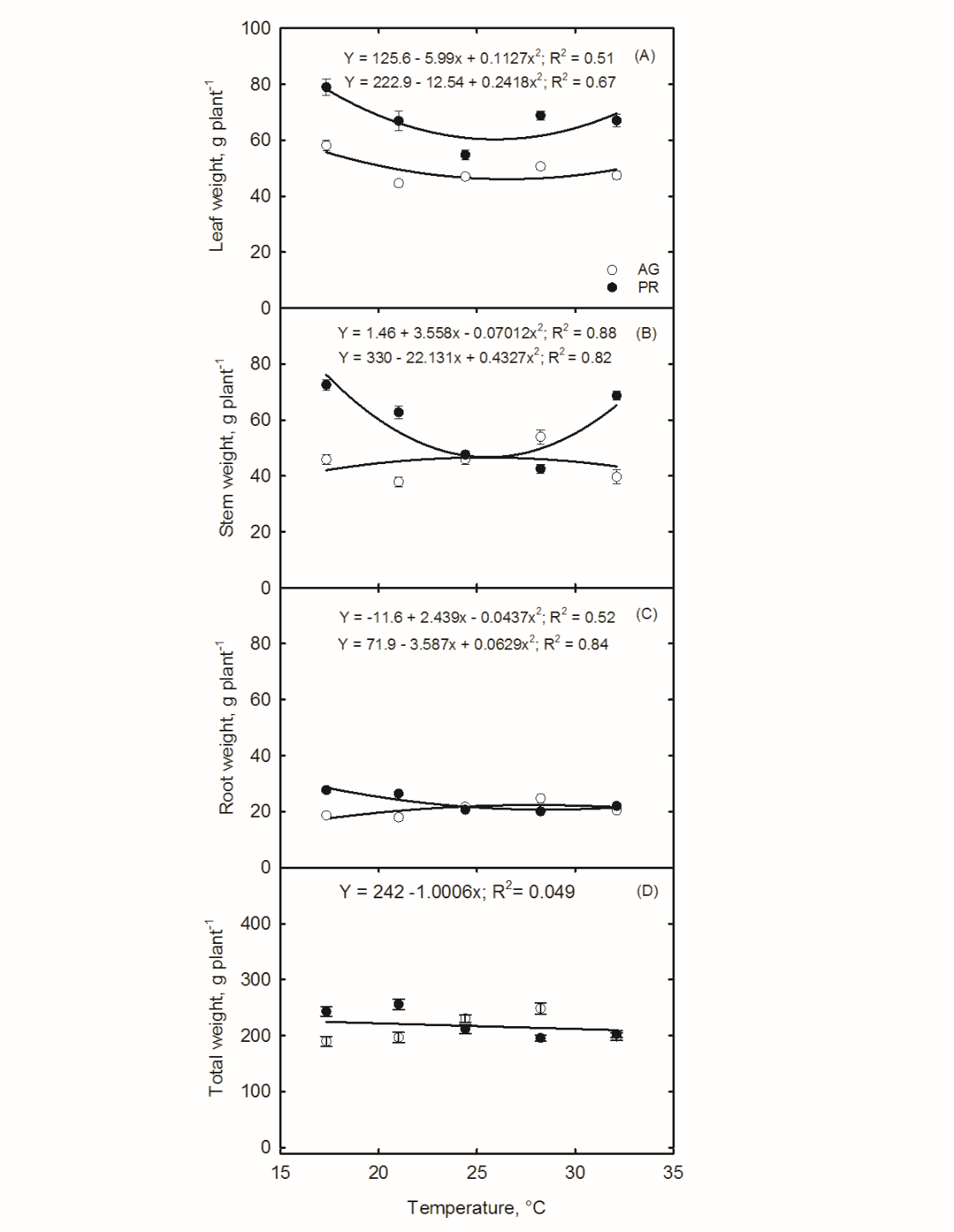


**SUPPLEMENTARY FIGURE** **2 |** Temperature effects on (A) leaf weight, (B) stem weight, (C) root weight, and (D) total dry weight of soybean cultivars, Asgrow AG 5332 (AG) with indeterminate, and Progeny P5333 RY (PR) with determinate growth habits, respectively. Measurements were taken at 120 days after sowing and 82 days after temperature treatment. Standard errors of the mean ± 12 observations are presented if the values are larger than the symbol size.
